# Which is the Digital Competence of Each Member of Educational Community to Use the Computer? Which Predictors Have a Greater Influence?

**DOI:** 10.1007/s10758-023-09646-w

**Published:** 2023-04-04

**Authors:** Francisco D. Guillén-Gámez, Ernesto Colomo-Magaña, Andrea Cívico-Ariza, Teresa Linde-Valenzuela

**Affiliations:** 1 Department of Didactics and School Organization, University of Málaga, Málaga, Spain; 2 Department of Theory and History of Education, Faculty of Education, University of Málaga, Blvr. Louis Pasteur, 25, 29010 Málaga, Spain

**Keywords:** Digital competence, ICT, Technology, Computers, Educational community

## Abstract

Since the COVID-19 pandemic, the use of digital resources and virtual platforms is even more essential to continue the educational process, either in person or online, affecting all the members involved in the teaching–learning process of the students. Therefore, this study is aimed: (1) to know and compare the digital competence of the agents that are the main integrators of the educational community (parents, teachers, students) about the use of the computers, according to gender and educational stage (Pre-school, Primary and Secondary Education stages); and (2) to identify significant predictors that affect the acquisition of this competence. An ex-post-facto design was used with a sample of 786 participants from Andalusia (Spain). Research methods such as contrasts of means and multiple linear regression analysis were used. The results showed high average levels of basic digital competences for all the agents involved. A gender gap was found between mothers and fathers of students, with higher scores for the latter group. In addition, the use of videogames, the parents' academic background and the use of digital tablets or Google+ are the most significant predictors that affect the acquisition of this competence.

## Introduction

The state of emergency decreed in most countries (Virgili, 2021), made that educational communities (students, teachers and parents) had to deal with an unprecedented situation (Corell et al., 2021; Huber & Helm, 2020). This new scenario reflected the need and responsibility of the entire educational community to acquire new competences, including digital competences (Montaudon-Tomas et al., 2022), as digital resources and *learning management system* (LMS) were the only means teachers-students-parents had to continue teaching, as well as to interact and communicate among them (Qiu et al., 2021). In short, it is necessary for the entire educational community to acquire skills in the use of information and communication technologies (ICT) so that communication between all the agents involved in the educational process is as effective as possible (Bordalba & Bochaca, 2019.)

To achieve this, it was imperative to implement digital training of all agents: (1) for teachers, to facilitate technology-mediated learning for their students (López Belmonte et al., 2020; Sánchez-Cruzado et al., 2021) and to promote the online participation of families in the school (Linde-Valenzuela et al., 2019); (2) for parents, to maintain family-school communication safely, as well as to support learning processes from home; and (3) for students, to be able to continue their online educational process development (Supardi & Hakim, 2021), with a need for accompaniment and guidance (Mielgo-Conde et al., 2021).

The digital literacy of members of the school community may be affected by difficulties in accessing information and technological resources (Varela et al., 2020), generating inequalities reflected in a double exclusion, both social and digital (Devkota, 2021). This prevents citizens from exercising their rights and participating in a society largely governed by technology (Robles et al., 2021) and has become an issue of exogenous origin the solution of which requires a structural approach.

The effect of the digital gap on social exclusion (Gómez-Trigueros et al., 2023), as well as the influence of the gender digital gap as the second digital gap (Garrido et al., 2019), is an additional barrier to overcome for the comprehensive development of citizenship in the twenty-first century (Joiner et al., 2015). As stated by Mariscal et al. (2019), "there is still a stark and pervasive gender inequality in terms of access, ownership of digital devices, digital fluency as well as the capacity to make meaningful use of the access to technology". The gender of people can be a relevant factor in learning (Yu, 2021), and therefore, in the development of digital competence (Bustamante-Barreto et al., 2022; Wongwatkit et al., 2020). Furthermore, Ortega and Gómez (2019) affirm that more and more authors through their studies tend to consider the digital gender gap as a problem of great importance, together with the difficulties in accessing and using ICT and the development of basic digital skills.

Compared to the uncertainty as to when this new educational scenario will end, we need to analyse the current state of the digital competences of all the agents involved in the educational process in this educational moment which was online, in order to find out if they are suitably trained and adapted to adjust to what is, for many of them, a new methodology. With this, the authors highlight the original contribution of this study: it is true that in the scientific literature there are many studies that analyze the digital competence of teachers at different educational stages, as well as the skills of students. However, there are hardly any studies that analyze and compare the digital competence of these two agents jointly in all the compulsory educational stages (Preschool Education, Primary Education and Secondary Education), adding extra value to the incorporation of another educational agent which has hardly been studied, the families of the students. In addition, it is studied in depth which predictors significantly affect the agents of each educational stage and for each gender in particular. In this way, we manage to have a complete and holistic vision of the digital competences of the entire educational community.

Faced with these new educational demands, the authors ask ourselves: Does the educational community (teachers, students and the parents of their students) have basic training in digital competence? Is gender still a major digital divide? Are there predictors that influence its development?

## Related Works

This section is classified into five sections. In the first three, related works are described on the level of digital competence of the agents that make up the educational community (teachers, students and their fathers and mothers), for various educational stages (Preschool, Primary and Secondary Education). The fourth section is focused on those factors which affect the acquisition of digital skills. Finally, it is reflected on what has been investigated on this matter so far, and the contribution of the authors with this study.

### Digital Competence of Teachers

The use of technology for e-learning ever since the pandemic started has highlighted the need for digital *competence* among teachers (Trust & Walhen, 2021). The accelerated pace at which technology had to be used left teachers feeling ill-prepared for effective online teaching (Novoa-Castillo & Sánchez-Aguirre, 2020).

At the Pre-school Education stage, Gjelaj et al. (2020) conducted a qualitative study with eight randomly selected teachers from Kosovo. The teachers stated that their education system did not support any digital technology learning tools, so they have to make do with little or no digital educational training. Similar results were found by Tileva (2020), Luo et al. (2021) and Casillas et al., (2020). In contrast, Otterborn et al. (2019) carried out an analysis of the use of digital resources (tablets) by 327 Pre-school Education teachers belonging to Sweden, showing a high degree of use, with activities aimed at generic and social skills. Cabero-Almenara et al. (2021) analysed the digital competence of in-service teachers (*n* = 1194) belonging to Spain. The results displayed a negative bidirectional relationship in digital competence from the Pre-school Education stage (with higher scores) regarding higher stages (with lower scores), with a higher success rate among female teachers. Similar results were found by Arouri et al. (2020) and Pozo et al. (2020), although contradictory to Guillen-Gamez et al., (2021a).

At the Primary Education stage, Guillén-Gámez and Ramos (2021) analysed the digital skills of 115 Spanish teachers currently active who were specialised in the subject of music. The results showed that teachers had a novice-expert profile, in addition to gender differences regarding the use of electronic devices by female teachers. With more unfavourable results, Obaydullah and Rahim (2019) found in a sample of 40 teachers from Bangladesh, that they were not prepared to integrate ICT in the classroom, identifying the lack of digital resources as one of the main causes. However, contradictory results were found by Roussinos and Jimoyiannis (2019) when they stated that a sample of 399 teachers belonging to Greek had a good level of TPAK (technology with pedagogy) knowledge, with male teachers showing higher competency.

At the secondary school level, Mailizar and Fan (2020) analysed the digital competences of 341 mathematics teachers from Indonesia. The results suggested that teachers had inadequate knowledge of using ICT for teaching mathematics. Similar findings were shown by Buabeng-Andoh (2019) as teachers were limited to basic and traditional activities such as searching for information or presenting in class, where female users displayed higher levels. Contradictory results were identified by Guillén-Gámez et al., (2021b) stating that a sample of 81 teachers in Madrid (Spain) had average levels of knowledge and use of digital resources, including Moodle, so significant differences in respect of gender (with males displaying a higher knowledge) were stated.

### Family Digital Competence

Parents are essential in supporting the teaching–learning process of their children and this is why digital communication with teachers must be constant and fluid (Franky & Chiappe, 2018). In this context, Romero et al. (2021) offers a series of proposals aimed at families regarding the education of their children in relation to media and informational education, how to supervise and teach responsible use of the internet or create spaces for communication between families and minors on digital media. From the school environment, it is important to promote the virtual participation of families in the educational centre and facilitate family-school digital communication (Antolín et al., 2018), as well as raise awareness of the appropriate digital tools for two-way communication (Maciá & Garreta, 2019).

Considering previous studies, we found that Tomczyk (2018) analysed the digital competence of 183 mothers and 76 fathers from the city of Tarnów (Poland) on the safe use of electronic resources. The results showed low levels of digital competence on copyright and safe interactions with other Internet users (including sexting). Similar results were published several years later by Tomczyk and Potyrała (2021). In a similar context, Real et al. (2018) studied the competences of 43 fathers and 229 mothers belonging to USA, on the use of smartphones for educational purposes, with findings that stated that a part of the sample had difficulties in connecting via Wi-Fi or did not feel comfortable using technology. In the same vein, Soldatovaa and Rasskazovab (2014) in their study carried out on Russian territory found that the lack of technology skills within families needed to be addressed with educational programmes; although children were also found to be good guides in their parents' digital competence (Nelissen & Van den Bulck, 2018). Nevertheless, none of these previous studies made a comparison by gender. Along these lines, Bartholomew et al. (2012) analysed the self-efficacy of 154 mothers and 150 fathers belonging to the Midwestern U.S., on Facebook use, which stated significant differences in favour of the female gender. Yaman et al. (2021), in turn, collected a sample of Turkey 3930 mothers and 604 fathers, finding that, in respect of digital skills for writing correctly when using the Internet, mothers paid more attention to grammatical rules than fathers.

### The Digital Competence of Students

Today's students are portrayed by the media as "digital natives" (Creighton, 2018). This new generation of students is defined as emotionally attached users of ICT (Turner, 2015), mainly for leisure purposes (Allaby, 2018; Aranda et al., 2019). However, this perspective was lately criticised by different researchers (Flynn, 2021; Kirschner & van Merriënboer, 2013), as perhaps their competence levels are not focused on educational technology (Judd, 2018).

In terms of Primary School students, Moreira et al., (2018, p. 249) stated that "it is a generation familiar with digital technology, which demands continuous school use of ICT", where levels of digital competence are adequate (Porat et al. (2018). However, other authors are not entirely convinced by these statements (Koutropoulos, 2021), because digital competence does not come naturally or is already acquired but needs to be developed in educational contexts. For example, the PISA report (OECD, 2011) highlighted the digital shortcomings of young people. Similar results were found by Paredes-Labra et al. (2019) who analysed the digital skills of 206 students from Madrid (Spain), in their final year of primary education, showing a low proficiency in information search, content creation and problem-solving skills using devices.

Regarding Secondary Education, Kaarakainen et al. (2018) assessed the basic, advanced and professional ICT digital skills of 3,159 Secondary Education students and 626 teachers from Finland. Both types of participants achieved a medium score on basic skills, medium–low on advanced skills, and very low on professional skills. By gender, both students and teachers scored higher if they were male. With similar results, Hatlevik and Christophersen (2013) analysed the digital skills of a sample of 4,087 Secondary School students from Norway, obtaining an average level, although they found no differences by gender. Perhaps one explanation for these results can be found in the work of McCahey et al. (2021), whose research showed that students (*n* = 14,530) did not frequently use ICT outside school for schoolwork, but rather for leisure activities.

### Factors Affecting Digital Competence

Many authors have endeavoured to link predictors that significantly affect the acquisition of digital competence, such as school infrastructure and Internet access (Lucas et al., 2021), or personal variables like age (Valdez et al., 2021) with a negative relationship, although other authors found no correlation (Tondeur et al., 2018).

Other studies have shown that the use of digital resources increases both the acquisition of new skills (Csordás, 2020) and has a direct effect on academic performance (Mehrvarz et al., 2021). For example, Hernández-Martín et al. (2021) found that students who use social networks on a daily basis have better digital skills than those who use them only three or four times a week, so there is a positive relationship between the intensity and use of ICT and student learning in terms of their digital skills (Martinez-Lopez et al., 2020).

Another understudied factor related to digital competence is the educational background of the parents, which may affect their children's education (Idris et al., 2020). Some studies have shown that the parents' beliefs and use of digital media is closely related to their children's self-efficacy and digital skills (Hammer et al., 2021), so educational policies must be put in place to promote the digital competence of parents with little education (Odoh et al., 2017).

Finally, this competence can be improved by the use of videogames (de Prado, 2018; Tulowitzki et al., 2019). This is, however, not a resource frequently used in the educational process, perhaps due to shortcomings in the training of educators, lack of devices (Del Moral & Fernández, 2015) or even false myths surrounding them (de Prado, 2017). For example, Marín-Díaz et al. (2015) found in their study that students learned better through collaboration and teamwork when using educational videogames.

### Reflection of the State of the Art and Study Contribution

After this reflective view of the state of the art, it is necessary to question whether the educational community is sufficiently prepared in this COVID era to face a teaching–learning process marked by the use of ICT through virtual teaching. The literature reveals a lack of studies that jointly analyse the digital competences of the different agents involved in this process (students, teachers and parents), as well as the identification of predictors impairing their acquisition. Therefore, the objectives of this study are:

O1. To find out and compare the level of digital competence of the members of the educational community (parents, teachers and students), at each educational stage (Pre-school, Primary and Secondary Education) by gender.

O2. To identify significant predictors in those groups that present significant differences by gender.

## Method

### Design and Participants

An ex-post-facto design with purposive sampling was used. Data collection was carried out in the second semester of the 2019/2020 academic year, with a total of 786 participants from Andalusia (Spain). For the Early Childhood Education stage, data was collected from the teachers who taught in the last year of this stage, as well as the relatives of the students enrolled in this course. The collection of data from students at this stage (approximately 5 years) was not taken into consideration because their level of reasoning to carry out a survey is not yet sufficiently developed. For the Primary Education Stage, students enrolled in fifth and sixth grade were used; and for the Secondary Education stage, first- and second-year students were used. In addition, data was collected from the families of the same students who had filled out the survey, both in Primary and Secondary Education. At all times, the confidentiality of the data was maintained, assuring the participants the privacy of their responses. Table 1 shows the distribution in percentages and average age of each type of educational agent.

**Table 1:** Sample distribution

Sample	Male		Female	
	Frequency	Age	Frequency	Age
*Pre-school*				
Teachers	33.8% (*n* = 26)	34.92 ± 5.02	66.2% (*n* = 51)	33.35 ± 5.55
Families	39.7% (*n* = 25)	32.04 ± 5.65	60.3% (*n* = 38)	29.94 ± 7.34
*Primary*				
Students	38.3% (*n* = 54)	11.43 ± 0.24	61.7% (*n* = 87)	10.72 ± 0.91
Teachers	37.8% (*n* = 28)	50.48 ± 29.71	62.2% (*n* = 46)	44.87 ± 23.71
Families	41.6% (*n* = 52)	38.62 ± 5.77	58.4% (*n* = 73)	34.14 ± 13.21
*Secondary*				
Students	49.6% (*n* = 57)	13.02 ± 0.98	50.4% (*n* = 58)	12.08 ± 1.01
Teachers	53.9% (*n* = 41)	46.38 ± 14.82	46.1% (*n* = 35)	44.31 ± 10.15
Families	37.9% (*n* = 36)	38.99 ± 16.65	62.1% (*n* = 59)	38.31 ± 14.85

### Instrument

To achieve the objective of measuring the level of digital competence, an ad-hoc questionnaire on the use of digital applications was developed and created by the authors themselves. There are many instruments focused on the digital competences of teachers in relation to the pedagogical competence, such as the DigCompEdu model (Digital Competence of Educators) prepared by Redecker (2017), as well as instruments focused on citizen digital competences with models such as DigCom. 2.1 (The Digital Competence Framework for Citizens) by Carretero (2017). However, we considered creating our own instrument which would be equally valid for both students, their families and their teachers, since the first two groups have probably not developed in pedagogical skills compared to teachers, who in their academic training has been formed. Therefore, we consider creating a battery of basic items which can measure the same for the three agents involved.

The instrument was composed of four dimensions with a total of 25 items: DIM. (A) Hardware and Operating System Computer Competence; DIM. (B) Computer competence on Office software; DIM. (C) Information competence on the Internet; DIM. (D) digital literacy. A seven-point Likert scale was employed to measure the items, where a value of 1 was associated with a low/very low digital competence, and a value of 7 was associated with a high/very high digital competence action.

The validity of the instrument was ascertained through two analyses. First, an exploratory factor analysis (EFA) was carried out with IBM SPSS V.24 software. In its application, the maximum likelihood method by oblimin rotation was selected. Throughout the process, several criteria were taken into consideration with the purpose of selecting those items which create a satisfactory model: those items with symmetry and kurtosis values in the range ± 1 were selected (Pérez & Medrano, 2010), it was also discarded those items with coefficients less than 0.4 in relation to the homogeneity index, the selection of factors with eigenvalues greater than one (Gümüş & Kuku, 2022).

The Kaiser–Meyer–Olkin index (KMO) was very satisfactory, with a value of 0.952, as was Bartlet's test of sphericity, which was significant (X2 = 8204.075; *p* < 0.05). These values are within the thresholds recommended by Field (2013), Worthington & Whittaker (2006) and Kalaycı (2010). The latent structure showed that the four factors explained 71.61% of the instrument total variance. Specifically, each factor explained the following percentages: DIM. A (55.56%), DIM. B (7.13%), DIM. C (5.06%), and DIM. D (3.86%).

**Fig. 1:**
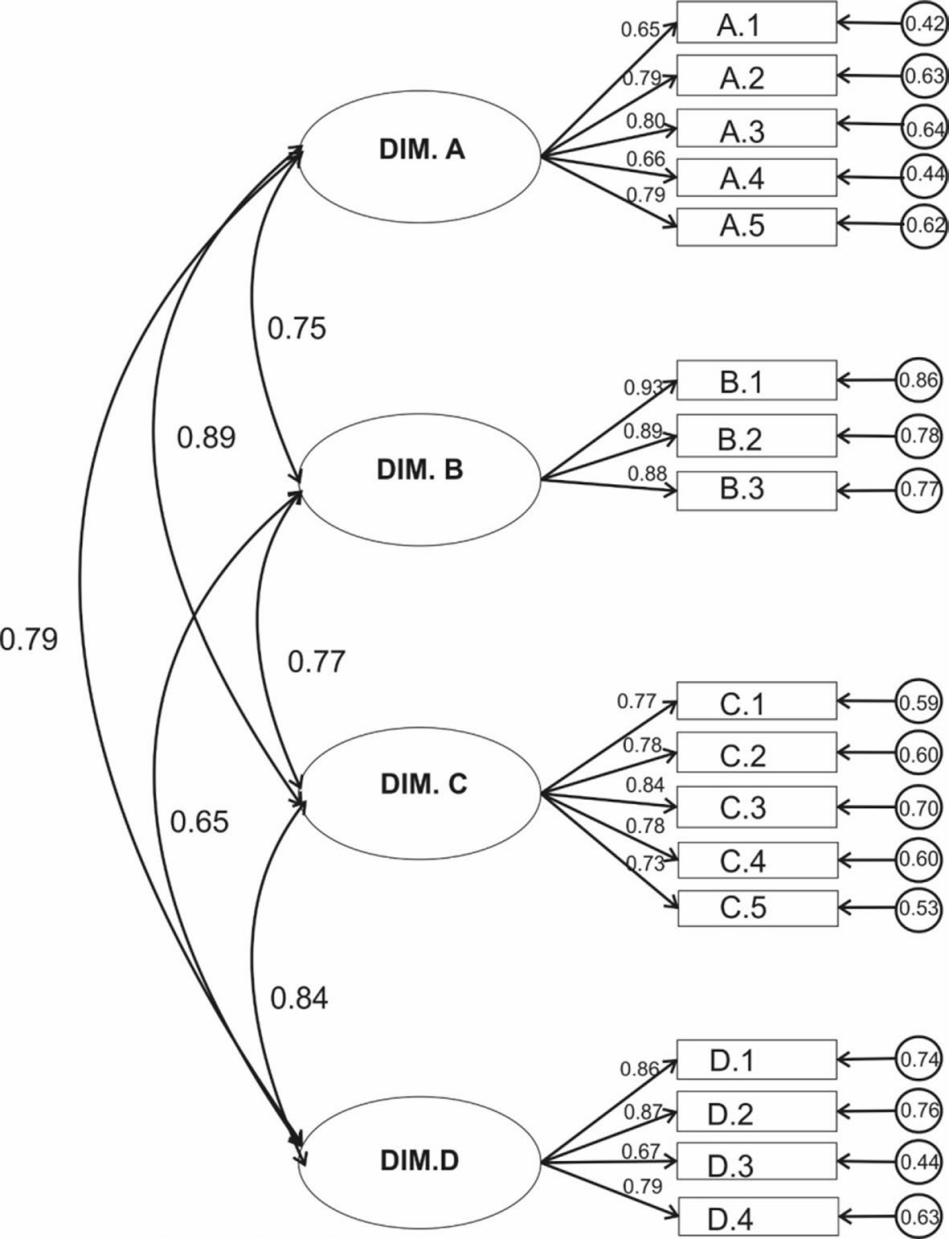
Model fit (CFA)

Secondly, the confirmatory factor analysis (CFA) reduced the number of final items to 17. This analysis was developed using AMOS V.24 software, taking into consideration the recommendations provided by Hu and Bentler (1999) on the value ranges that the following indices should have: minimum discrepancy (CMIN/df = 3.898), the goodness-of-fit index (GFI = 0.904), the parsimony goodness-of-fit index (PGFI = 0. 724), the normalised fit index (NFI = 0.932), the parsimony normalised fit index (PNFI = 0.747), the incremental fit index (IFI = 0.949), the Tucker-Lewis index (TLI = 0.936) and root mean square error of approximation (RMSEA = 0.067). Finally, the reliability of the instrument obtained very satisfactory values: Cronbach's Alpha (*α* = 0.949); McDonald's Omega (*α* = 0.993). The structural equation model can be seen in Fig. 1. In Annex 1 you can see the description of the items in the questionnaire.

## Results

### Descriptive and Comparative Analysis on Digital Competence According to Gender and Educational Stage

Table 2 classifies the participants by educational stage and group to which they belong, according to their gender. The Kolmogorov–Smirnov test (*p*. > 0.05) and the Levene *t*-Student tests were applied. Cohen's d was calculated to determine the effect size. According to Rosenthal et al. (1994), values of approximately 0.2, 0.5 and 0.8 indicate small, medium and large effects, respectively. Concerning the Pre-school Education stage, it can be observed that teachers have a medium–high level of literacy, being slightly lower in females (*M* = 4.31) than males (*M* = 4.46). Nevertheless, there were no significant differences between both sexes (*p*. = 0.517). Regarding the families, they display a low level of literacy, slightly higher in males (*M* = 2.08), with significant differences between their scores (*p*. = 0.011) with a large effect size (*d* = 0.862). At the Primary Education stage, students display a high average level of digital competence, slightly lower in females, although there are no significant differences between the two (*p*. = 0.058). As to teachers, there was a greater difference between both sexes, with lower results for females (*M* = 4.30) than for males (*M* = 5.45). However, there were no differences between them (*p*. = 0.755). In the group of families, there was a slight difference in the scores of women (*M* = 3.24) compared to men (*M* = 3.63), with significant differences between the two (*p*. = 0.001) and a medium effect size (*d* = 0.601). At the Secondary Education stage, a high average literacy level was observed for both sexes, being lower in females (*M* = 4.41) compared to males (*M* = 4.47), with no significant differences between them (*p*. = 0.648). A similar score was observed for teachers, with a few tenths of a point lower in women (*M* = 4.74) compared to men (*M* = 4.81), with no significant differences between them (*p*. = 0.791). In the family group, a lower score was observed in women (*M* = 2.85) compared to men (*M* = 4.02), with significant differences between them (*P*. = 0.001) with a large effect size (*d* = 0.817).

**Table 2:** Difference of agreed means to gender and group

Stage	Group	Sex	Score	Levene	t-Student		
					t	Sig	Effect Size **d**
Pre-school							
	Teaching staff	Female	4.31 ± 0.95	.060	− 0.651	0.517	–
		Male	4.46 ± 0.84				
	Families	Female	1.68 ± 1.47	.011	− 3.349	0.001	0.862
		Male	2.08 ± 0.77				
Primary							
	Students	Female	4.18 ± 1.08	.874	− 1.135	0.058	–
		Male	4.47 ± 1.04				
	Teaching staff	Female	4.20 ± 2.07	.221	0.314	0.755	–
		Male	5.45 ± 0.47				
	Families	Female	3.04 ± 1.27	0.001	− 3.308	0.001	0.601
		Male	3.63 ± 0.73				
Secondary							
	Students	Female	4.41 ± 1.20	0.587	0.458	0.648	–
		Male	4.31 ± 1.20				
	Teaching staff	Female	4.74 ± 1.02	0.669	− 0.266	0.791	–
		Male	4.81 ± 1.22				
	Families	Female	2.85 ± 1.72	0.001	− 3.862	0.001	0.817
		Male	4.02 ± 0.99				

Considering that only the group of parents has statistically significant differences between both sexes, we now analyse for this group and for each educational stage, how a series of predictor variables influences the overall level of digital competence through a multiple linear regression model (MLR).

### Predictive Analytics in Digital Competence of Parents

Table 3 lists the predictors analysed for the whole level of digital competence, or the group of fathers and mothers. Variables IV1 to IV 7 have been coded as ordinal variables, with a 7-point Likert scale, where value 1 is associated with the label "I use it not very often" and value 7 with the label (I use it very often). The educational level variable was coded as a polytomous with seven categories (1-no studies; 2-primary studies; 3-secondary studies; 4-bachelor's degree; 5-university degree; 6-Master's degree; and 7-doctorate). Finally, the variable age was categorised as a ratio variable.

**Table 3:** Description of variables

ID	Description
IV 1	How often do you use educational videogames with your child?
IV 2	How often do you use entertainment videogames with your child?
IV 3	How often do you use tablets to help your child with homework?
IV 4	How often do you use WhatsApp family groups?
IV 5	How often do you consult the teacher's blog?
IV 6	How often do you use e-Books?
IV 7	How often do you use Google digital apps to help with your child's homework?
IV 8	What level of education do you have?
IV 9	How old are you?

The first step was to check the assumptions of this type of statistical technique. The assumptions of normality, independence and multicollinearity of the residuals were met. The coefficients of the Durbin-Watson statistic fell within the recommended values (threshold 1.5–2.5), whereas the tolerance values were greater than 0.6 and the variance inflation factor (VIF) was below 10 (Ghani & Ahmad, 2010).

Table 4 shows the standardised weights for the analysed predictors of the overall level of digital competence for parents who had children at the Pre-school Education stage. It can be seen that the female gender had only one significant predictor, age, with a negative direction. This data explained 16.80% of the variance in terms of digital competence level; however, the model was not significant (*p*. < 0.05). For the male gender, it is observed that participating in educational and entertainment (non-educational) games significantly influences the level of digital competence, positively and negatively, respectively, explaining 21.80% of the true scores in the overall digital competence level. Therefore, the equations of the regression model can be seen as follows, taking into account the standardised coefficients in the same unit:

**Table 4:** Coefficients for parents in Pre-school Education stage

	Female (*R*^2^) = 16.80%			Male (*R*^2^) = 21.80%		
	*β*	*t*	*p*	*β*	*t*	*p*
Constant	2.782	1.215	.235	6.580	5.388	.001*
VI 1	.374	1.483	.150	.571	3.887	.002
VI 2	− .070	− .285	.778	− .563	− 3.270	.006
VI 3	.200	.633	.533	− .493	− 1.038	.318
VI 4	− .087	− .516	.610	− .256	− .981	.344
VI 5	.009	.040	.968	− .236	− .843	.414
VI 6	− .083	− .402	.691	− .286	− .803	.436
VI 7	.148	.443	.661	.786	1.429	.176
VI 8	.296	1.090	.286	.016	.080	.938
VI 9	− .253	− 2.844	.009*	− .104	− .835	.419

*Significance level at 95%. *β*: standardised coefficient

- **Mothers** = 2.782 − 0.253*Age.
- **Fathers** = 6.580 + 0.571*Educational −0.563*Entertainment.

For parents of Primary School students (Table 5), it is observed that playing both types of videogames (educational and entertainment) with their children has a significant influence, with a positive and negative direction, respectively. For both genders, the level of education acquired also has a significant positive influence. The use of tablet apps and Google apps to help a child with its homework has a positive influence only on the female gender. These predictors explain 24.56% of the variance in the true global digital competence scores for this gender. The equations with the standardised weights are:

**Table 5:** Coefficients for parents of primary school students

	Female (*R*^2^) = 24.56%			Male (*R*^2^) 39.20%		
	*β*	*t*	*p*	*β*	*t*	*p*
Constant	1.884	2.548	.013	3.946	.529	.001
VI 1	.207	2.171	.034*	.203	2.040	.048*
VI 2	− .193	− 2.255	.028*	− .445	− 4.541	.001*
VI 3	.225	3.745	.001*	.094	1.029	.309
VI 4	.094	1.695	.095	.026	.315	.754
VI 5	.067	.899	.372	.091	1.129	.256
VI 6	− .037	− .490	.626	.081	1.027	.310
VI 7	.192	2.452	.017*	.140	1.402	.168
VI 8	.267	3.725	.000*	.233	2.183	.035*
VI 9	− .093	− 1.867	.067	.015	.181	.857

^*^ Significance level at 95%. β: standardised coefficient

- **Mother** = 1.884 + 0.207*Educational game − 0.193*Entertainment game + 0.225*Tablets + 0.192*Google 0.267*Training.
- **Father** = 3.946 + 0.203* Educational game—0.445* Entertainment game + 0.233* Training.

For parents of Secondary Education students (Table 6), similar and significant results were found with respect to parents of Primary Education students, and also partially similar to those of Infant Education pupils, in relation to participation in educational and entertainment videogames. In addition, the use of digital applications offered by Google+ , as well as the educational level of both parents significantly predict the level of digital competence, with a positive direction. Only the use of digital tablets turns out to be a significant predictor for the female gender. The model for the female gender was found to explain 24.56% of the true variance for the female gender, while for the male gender it was higher, with a percentage of 39.20%. The equations were as follows:

**Table 6:** Coefficients for parents of Secondary School students

	Female (*R*^2^) = 24.56%			Male (*R*^2^) 39.20%		
	*β*	*t*	*p*	*β*	*t*	*p*
Constant	2.979	3.318	.002	3.831	4.726	.001
VI 1	.402	3.598	.001*	.356	2.593	.015*
VI 2	− .277	− 2.493	.016*	− .332	− 2.678	.012*
VI 3	.126	1.994	.042*	− .066	− .832	.413
VI 4	− .080	− 1.631	.109	− .090	− 1.128	.269
VI 5	.647	1.097	.278	.046	− .978	.089
VI 6	− .606	− 1.029	.309	.083	1.113	.276
VI 7	.286	1.840	.044*	.164	1.691	.039*
VI 8	.139	2.080	.043*	.397	3.023	.005*
VI 9	− .055	− 1.140	.260	.052	.587	.562

*Significance level at 95%. *β*: standardised coefficient

- **Mother** = 2.979 + 0.402* Educational game −0.277* Entertainment game + 0.126Tabletas + 0.286Google + 0.139* Training.
- **Father** = 3.831 + 0.356* Educational game −0.332* Entertainment game + 0.164*Google + 0.397* Training.

## Discussion

In the context of online training derived from SARS-CoV-2, this study has analysed the level of digital competence of members of the educational community, considering gender as a factor of analysis (O1). In addition, predictors of this level have been identified for agents with significant differences according to gender (O2).

In relation to the first objective and focusing on the group of students, the results at the Primary Education stage point to an average level of digital competence, similar to what was found in the work of Porat et al. (2018). Our results differ from those of Paredes-Labra et al. (2019), where low proficiency was found in actions such as content creation or information search. Although the male gender had higher proficiency, no significant differences were found. At the Secondary Education stage, students had an intermediate level, as in the work of Kaarakainen et al. (2018), where most students had basic skills and very few were able to demonstrate more advanced or professional skills. Furthermore, no gender differences were found, as in the study by Hatlevic and Christophersen (2013). Most of these studies find medium-basic levels, suggesting that this may be a consequence of the age variable, where the use of technologies is of a playful rather than formative nature for students at this age (McCahey et al., 2021).

Focusing on teachers, we detect a medium–high level in Pre-school Education, contrary to other studies (Casillas et al., 2020; Gjelaj et al., 2020; Luo et al., 2021; Tileva, 2020), where males scored higher than females, which also differs from the results of Arouri et al. (2020) and Cabero-Almenara et al. (2021). At the Primary Education stage, both genders show a medium–high level of digital competence, in contrast to the findings of Obaydullah and Rahim (2019) where the lack of preparation to integrate ICT in a didactic way is highlighted. As to gender, no significant differences were found, although male teachers had stronger digital skills, coinciding with the results of Roussinos and Jimoyiannis (2019), although these differ from those of Guillén-Gámez and Ramos (2021), where the female gender was found to have greater digital skills in the use of electronic devices. At the Secondary Education stage, both genders obtained medium–high scores, contradicting the work of Buabeng-Andoh (2019) and Mailizar and Fan (2020) where teachers had little knowledge of ICT beyond searching for information. The scores are higher in the male gender, coinciding with the work of Guillén-Gámez et al., (2021b) but contradict the findings of Buabeng-Andoh (2019), albeit without significant differences. In view of these results, it is likely that the differences found between teachers at the different educational stages (with lower scores in Early Childhood Education) are not due to a lack of knowledge about the use and management of ICT resources, but rather to the very nature of the educational stage and its recipients. This is what has a major effect on the poor use and acquisition of this competence.

As for fathers and mothers, we found a low and medium–low level of competence in the different educational stages, coinciding with the results of Real et al. (2018), Tomczyk (2018), and Tomczyk and Potyrala (2021). It is observed that the competence of fathers always exceeds that of the mothers. Significant differences were found at all educational levels. Although there is no history of gender studies, these results contradict studies such as Bartholomew et al. (2012) or Yaman et al. (2021), where mothers showed better competence in the use of social networks and in writing in digital media. These low levels, compared to those of their children and teachers, underline the need to implement training programmes with families (Soldatovaa & Rasskazovab, 2014), as well as to promote the use of technologies guided by their children (Nelissen & Van den Bulck, 2018) where the latter can be guides and mediators of the main emerging technologies that are being integrated into society.

In relation to the second objective and the identification of predictors of parents' digital skills, it was determined that age only affects mothers, negatively, at the Pre-school Education stage. This is in line with the study by Valdez et al., (2021) and in contrast to Tondeur et al. (2018), where no correlation was found. Another significant predictor (for both genders) was the use of videogames, except in mothers in Pre-school Education, where their use of these games in an educational way makes them develop more favourable skills compared to playing them or using them for entertainment. This fact coincides with the work of Marín-Díaz et al. (2015), de Prado (2018) and Tulowitzki et al. (2019), where the use of videogames reflects an improvement in digital skills. These results suggest that, if parents use educational videogames in their children's education, they would probably not only be improving the digital skills of their children, but would also be improving and developing their other skills such as creativity, memory, languages, teamwork and problem solving. However, these data should be reflected upon and taken into account with caution, and it is vitally important to carry out further research in this line of work.

Another significant predictor was the use of digital resources. For females, the use of digital tablets and Google+ applications, the latter also relevant for males, was found to have a direct effect on their digital skills, in line with the study by Csordás (2020). Furthermore, the educational level of both father and mother was found to be significantly related to their digital skills, mainly at higher educational stages. These results are in line with those of Mehrvarz et al. (2021), and suggest that the level of academic training that these individuals have gained over time through formal education is closely related to the acquisition of skills, including digital skills.

## Conclusions and Future Work

Online teaching in the different educational stages proved to be the solution to the COVID-19 pandemic. Thus, the use of technology has become mandatory for use in the educational process, involving the different agents (students, teachers and families). This study shows the importance of further developing digital competence in the educational community.

The results show that primary and secondary school students display a basic level of digital competence, this being slightly higher for teachers in all of the stages analysed (pre-school, primary and secondary). Parents, on the other hand, are at a low and medium–low level, being the agent with the lowest digital competence. Another main finding is that the male gender always shows a better level of digital competence than the female gender, being significant only in the case of families. For these, different predictive variables were analysed, therefore age only influences mothers with children in pre-school, with lower competence as their age increases. Videogames are a good predictor of the level of digital competence, having a positive impact if they are educational and a negative impact if they are used for entertainment. Other aspects such as educational level (parents with children in primary and secondary school), or the use of digital resources such as tablets (mothers with children in primary and secondary school) and Google digital applications (primary and secondary school for mothers and only secondary school for fathers) also predict to have a positive effect on their digital competence.

This research has different implications to highlight. Regarding the theory, the problem of the digital gap between the different agents that make up the educational process is highlighted, and it is necessary to expand the investigations to reduce these differences, paying special attention to families. At a methodological level, the detection of predictors on the improvement of digital competence guides how training processes should be designed so that they have a positive effect on learning. Regarding pedagogical practice, the need to reinforce and increase training in digital competence for all agents involved in the educational process is confirmed. Considering the influence of the analyzed predictors, providing technological devices to the different agents, along with the use of educational applications that promote motivation, are actions that can positively revert to improving digital competence.

The sample design has been found to constitute one of the limitations of this survey, due to its purposive nature and size. As the design is not random, the results cannot be entirely generalised, and for future work we would prefer a random sample with a larger number of participants, incorporating other interesting variables to be analysed (availability and access to technological resources, public or private educational centres, the employment of the parents, the years of experience of the teacher, among others). Another future research project would be to include the university stage, with special interest in education science degrees and teaching staff, analysing the development of digital competence in the preliminary training of future teachers. Furthermore, it would be interesting to implement digital competence training for the different agents in the educational community, carrying out a pretest–posttest study to track their progress, as well as to analyse the impact of increased use of technology-aided methodologies in the classroom, thus placing more focus on the students. Finally, another future work could be an intervention study where some parents are given training and the children’s online learning activity is compared with those whose parents haven’t been given training.

## Data Availability

The data is not shared because it is part of a doctoral thesis.

## Appendix 1 Questionnaire on Basic Digital Competence

**Table:**

DIM. A. Hardware and operating system computer competence
A.1 I have basic knowledge of how a computer works
A.2 I know how to connect a computer and its most common peripherals: printers, scanner, webcam
A.3 I know how to connect audio equipment, video cameras and digital photos to computers
A.4 I know how to use appropriate key combinations to get alphanumeric and punctuation marks from the keyboard
A.5 I am able to install and uninstall software on a computer
DIM. B. Computer competence on office software
B.1 I know how to use word processors (Word, OpenOffice, GDrive Docs)
B.2 I know how to use advanced techniques in word processors regarding style functions
B.3 I know how to use advanced techniques in word processors regarding insert functions (tables, graphics, SmartArt, links…)
DIM. C. Information competence on the internet
C1. I browse the Internet through the different links provided by the web pages that I visit
C.2 I am able to download with torrent programs, direct download, iTunes, or any other download platform
C.3 I can organize the information downloaded from the Internet, adding the pages that interest me to favorites, and classify them in subfolders under some ordering criteria
C.4 I am able to make video conferences (GMeet, Skype, Microsoft teams)
C.5 I am able to access, search and retrieve information using different forms of accessibility and formats
DIM. D. Digital literacy
D.1 I understand compatibility issues between computer hardware and software
D.2 I consider myself competent to know how to discriminate in most cases, email with viruses, junk or spam
D.3 I know how to use ICT tools and resources to manage and communicate personal and/or professional information
D.4 I know how to use word processor spell checkers to edit and proofread my work
